# Achiral bis-imine in combination with CoCl_2_: A remarkable effect on enantioselectivity of lipase-mediated acetylation of racemic secondary alcohol

**DOI:** 10.3762/bjoc.6.134

**Published:** 2010-12-10

**Authors:** K Arunkumar, M Appi Reddy, T Sravan Kumar, B Vijaya Kumar, K B Chandrasekhar, P Rajender Kumar, Manojit Pal

**Affiliations:** 1 Custom Pharmaceutical Services, Dr. Reddy’s Laboratories Limited, Bollaram Road Miyapur, Hyderabad 500 049, India; 2 Department of Chemistry, Jawaharlal Nehru Technological University of Anantapur, Anantapur 515002, Andhra Pradesh, India,; 3 Institute of Life Sciences, University of Hyderabad Campus, Gachibowli, Hyderabad 500 046, Andhra Pradesh, India

**Keywords:** acetylation, bis-imine, cobalt chloride, enantioselectivity, lipase

## Abstract

A bis-imine (prepared via a new FeCl_3_-based method) in combination with CoCl_2_ facilitated lipase-mediated acetylation of the (*R*)-isomer of a racemic benzylic secondary alcohol with 91% ee_s_. The methodology was used for the preparation of the known drug rivastigmine.

## Introduction

**Scheme 1:**
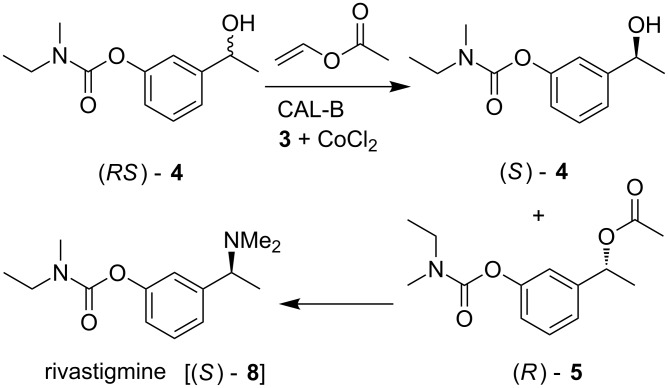
Lipase-catalyzed acetylation of racemic benzylic secondary alcohol [(*RS*)-**4**] and its application.

The development and use of newer synthetic methods for the stereoselective synthesis of chiral molecules have increased enormously in recent years especially in the chemical and pharmaceutical industry [1]. Biocatalysis, being an environmentally friendly process, has attracted particular attention for this purpose [2–6]. For example, high enantioselectivity was observed in lipase-mediated preparation of alcohols and amines [7–9]. These biocatalysts work under mild reaction conditions, and their immobilized forms, being stable in organic solvents, have allowed an easy separation of products and the potential recycling of the enzyme, thereby enhancing their economic viability [10–11]. Recently, we have observed that achiral bis-imines in combination with CoCl_2_ improved the enantioselectivity substantially in CAL-B (*Candida antarctica* lipase B**)** [12–13] mediated acetylation of a racemic secondary alcohol with vinyl acetate. Here we report our preliminary results on the synthesis and identification of a novel ligand for this process (Scheme 1) and its application in the preparation of the known drug rivastigmine [14]. While the uses of bis-imine/transition-metal complexes have been reported for the enantioselective synthesis of chiral compounds [15–19], their use as activators in an enzymatic reaction has not been previously explored.

## Results and Discussion

**Scheme 2:**
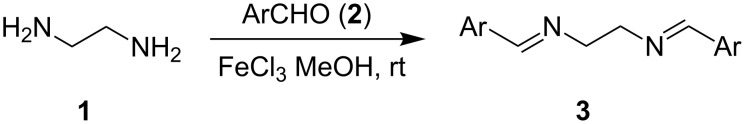
FeCl_3_-meditated synthesis of bis-imines.

The report that bis-imine/Cu(I)-complexes were able to promote the direct and enantioselective addition of imines to alkylacetylenes [15] prompted us to evaluate a variety of bis-imines in combination with CoCl_2_ [16] in the lipase-mediated acetylation of a benzylic secondary alcohols. Accordingly, a number of achiral bis-imines were prepared and used to generate the desired complex. While a number of methods have been reported to prepare Schiff bases by reacting an amine with a carbonyl compound [20–24], in our hands the reaction of 1,2-amines with aromatic aldehydes under these reaction conditions provided a mixture of mono and bis-Schiff bases. We therefore developed a new and efficient method for the preparation of bis-imines **3** by reacting ethane-1,2-diamine (**1**) with a number of aryl and heteroaryl aldehydes **2** in the presence of anhydrous FeCl_3_ (Scheme 2, Table 1). Aldehydes containing electron donating (e.g., methoxy, hydroxy, fluoro and bromo) or withdrawing groups (e.g., nitro) were found to be equally effective in terms of product yields. The reactions were completed within 30 min in most cases.

**Table 1:** FeCl_3_-meditated synthesis of bis-imines (**3**) from (hetero)aryl aldehydes (**2**).

Entry	Aldehyde (**2**)	Product (**3**)	Yield^a^ (%)	Reaction Time

1	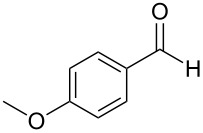 **2a**	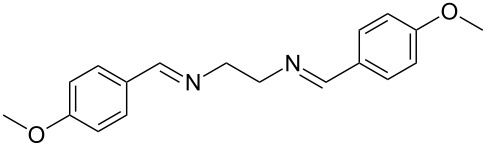 **3a**	90	0.5
2	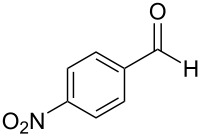 **2b**	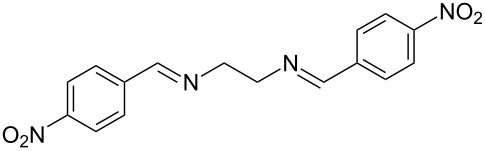 **3b**	90	0.15
3	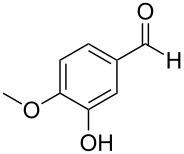 **2c**	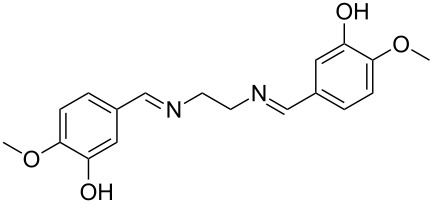 **3c**	88	0.5
4	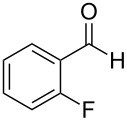 **2d**	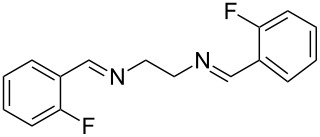 **3d**	80	0.5
5	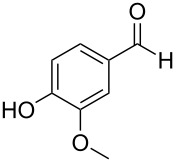 **2e**	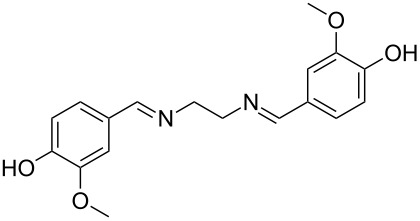 **3e**	80	0.5
6	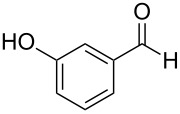 **2f**	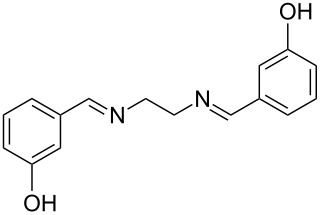 **3f**	88	0.5
7	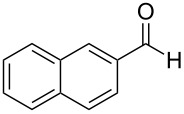 **2g**	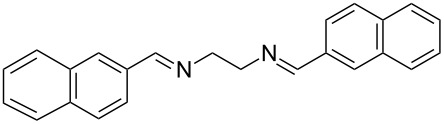 **3g**	80	0.5
8	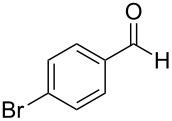 **2h**	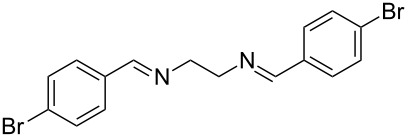 **3h**	85	0.5
9	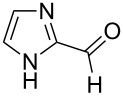 **2i**	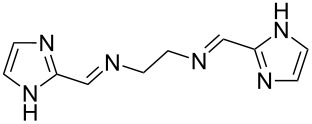 **3i**	90	0.5
10	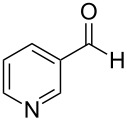 **2j**	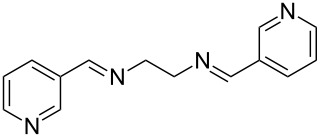 **3j**	90	0.5

^a^Isolated yield.

**Scheme 3:**
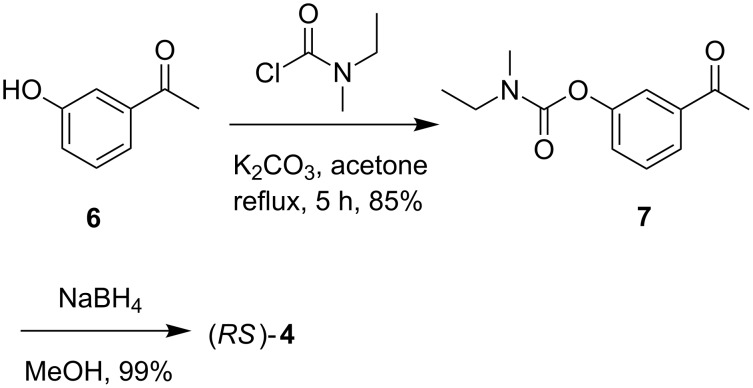
Preparation of racemic 3-(1-hydroxyethyl)phenyl ethyl(methyl)carbamate [(*RS*)-**4**]

All bis-imines prepared were then screened in combination with CoCl_2_ for CAL-B mediated acetylation of a racemic secondary alcohol. Thus, 3-(1-hydroxyethyl)phenyl ethyl(methyl)carbamate **[**(*RS*)-**4**], prepared via the reaction of 3-hydroxyacetophenone (**6**) with *N*-ethyl-*N*-methylcarbamoyl chloride followed by reduction with NaBH_4_ (Scheme 3), was selected for our purpose. Subsequently, the enzymatic processes were carried out and the isolated reaction mixture was analysed by chiral HPLC.

Initially, the CAL-B mediated acetylation of (*RS*)-**4** was carried out in the absence of any ligand and CoCl_2_. Vinyl acetate was used as a solvent as well as the acyl donor. No reaction was observed at room temperature even after 48 h. An increase in reaction temperature to 50–55 °C for 24 h facilitated the acetylation, however, the selectivity was not greater than 30%. In order to achieve better selectivity, we assessed the use of achiral bis*-*imines in combination with CoCl_2_ (Table 2). The reactions were complete within 10 h when diarylidene-ethane-1,2-diamines were used (entries 1–8, Table 2). While 35% enantiomeric excess was achieved in some of these cases (entries 2, 3 and 5, Table 2), the best results, however, were obtained with bis(heteroarylmethylene)ethane-1,2-diamines (entries 9 and 10, Table 2), especially **3i**. The bis-imine **3i** facilitated enantioselective acetylation of the (*R*)-isomer over the (*S*)-antipode with high enantiomeric excess (91% ee_s_) and yield (80%). The reaction was complete within 12 h. The absolute configuration of the resolved chiral alcohol and its acetate was in accordance with Kazlauskas’ rule [25] (see Supporting Information File 1 for optical rotation values).

**Table 2:** Screening of bis-imines as achiral ligands in CAL-B mediated acetylation of (*RS*)-**4** (step 1, Scheme 1)^a^.

Entry	Ligand **3**	Time (h)	ee_s_	ee_p_	Conversion^b^ (%)	*E*^c^

1	**3a**	10	0	0	0	0
2	**3b**	5	35	>99	~26	>200
3	**3c**	5	35	>99	~26	>200
4	**3d**	10	0	0	0	0
5	**3e**	5	35	>99	~26	>200
6	**3f**	5	0	0	0	0
7	**3g**	5	0	0	0	0
8	**3h**	5	0	0	0	0
9	**3i**	12	91	>99	~48	>500
10	**3j**	10	83	>99	~46	>300

^a^All the reactions were carried out at 1.0 g scale of (*RS*)-**4** with vinyl acetate (20 mL) as acyl donor, in the presence of CAL-B (150 mg), bis-imine (**3**, 0.3 mmol) and CoCl_2_ (0.3 mmol); ee_s_ = enantiomeric excess of substrate (the ee_s_ is mentioned as the enzyme is active with only one enantiomer) and ee_p_ = enantiomeric excess of product. Both ee_s_ and ee_p_ were determined by HPLC [column: chiralpak IC (250 x 4.6 mm, 5.0 μm), mobile phase A: 0.05% TFA in water, mobile phase B: *n*-hexane: IPA (80:20), concentration: 0.5 mg/mL, diluent: ethanol, run time: 30.0 min, temperature: 25 °C, flow: 1.0 mL/min, UV: 220 nm]. ^b^Conversion = ee_s_/(ee_s_ + ee_p_). ^c^*E* = {ln[ee_p_(1-ee_s_)]/(ee_p_+ee_s_)}/{ln[ee_p_(1 + ee_s_)]/(ee_p_ + ee_s_)}.

**Scheme 4:**
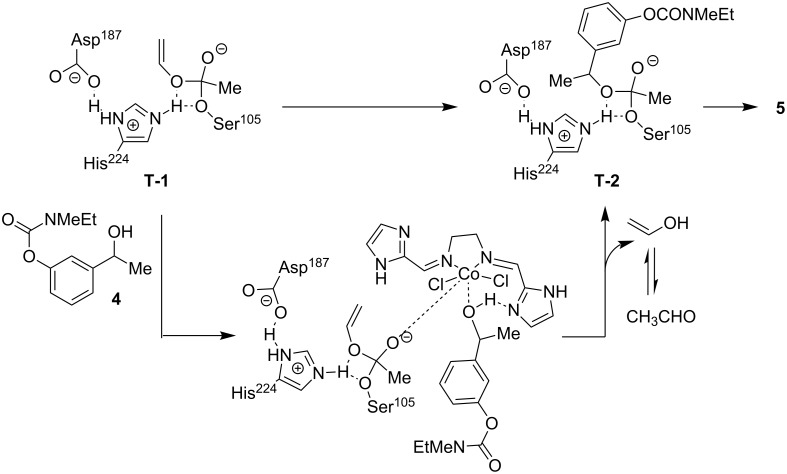
Proposed reaction mechanism of lipase-catalyzed acetylation of racemic alcohol **4**.

Mechanistically [12], the special H-bonding rearrangement of the “catalytic triad” (i.e., serine, histidine, and aspartate) at the active site of CAL-B increases the nucleophilicity of the serine residue. This then interacts with the carbonyl group of the vinyl acetate to form the “acyl-enzyme intermediate” **T-1** (Scheme 4) which finally transfers the acyl group to the substrate alcohol **4** via **T-2**, affording the desired product **5**. The CoCl_2_ in combination with **3i** perhaps forms a tight complex with **T-1** as well as **4** which facilitates the acyl transfer process (Scheme 4). However, the reason for selective acylation was not clearly understood. It was speculated that the orientation of the hydroxy group of the (*R*)-isomer was possibly in the proximal position of the acyl-transfer site and the imidazole moiety for proton abstraction.

**Scheme 5:**
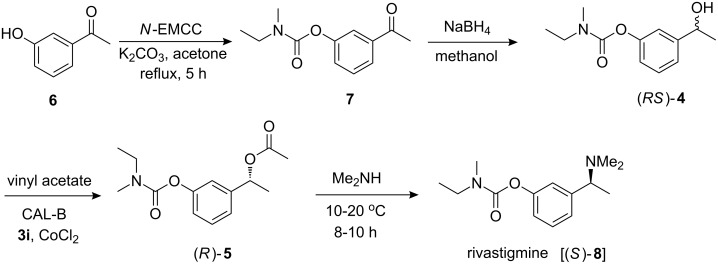
Complete synthesis of rivastigmine.

Finally, application of this methodology was demonstrated in preparing the well-known drug rivastigmine which has been used to treat mild to moderate dementia associated with Alzheimer’s and Parkinson’s disease. Thus the enantiopure acetate (*R*)-**5** was treated with excess of dimethylamine in toluene to afford the desired (*S*)-**8** [(*S*)-rivastigmine] in 60% yield (final step, Scheme 5). Notably, the earlier method for the synthesis of (*S*)-**8** involved asymmetric reduction of the ketone **6** to give the alcohol with the required chirality followed by mesylation and subsequent treatment with dimethylamine [26–27].

## Conclusion

We have developed a novel lipase-based method for acetylation of a benzylic secondary alcohol with high enantioselectivity and yield. The methodology involves the use of CoCl_2_ in combination with a bis-imine (prepared via a new FeCl_3_-based method) and its application has been demonstrated in preparing rivastigmine.

## Supporting Information

File 1 Experimental procedures and spectral data.
