# Supplementary material for: Achiral bis-imine in combination with CoCl2: A remarkable effect on enantioselectivity of lipase-mediated acetylation of racemic secondary alcohol
Source: Beilstein J Org Chem. 2010 Dec 10;6:1174–9. doi: 10.3762/bjoc.6.134 (PMC3002020; doi:10.3762/bjoc.6.134)
Supplement: File 1 — Experimental procedures and spectral data. [file Beilstein_J_Org_Chem-06-1174-s001.pdf]

Supporting Information

for

Achiral bis-imine in combination with $CoCl_2$: A remarkable effect on enantioselectivity of lipase-mediated acetylation of racemic secondary alcohol

K. Arunkumar[1,2], M. Appi Reddy[1], T. Shravan Kumar[1], B. Vijaya Kumar[1], K. B. Chandrashekar[2], P. Rajender Kumar[1], Manojit Pal[3,]*

Address: [1]Custom Pharmaceutical Services, Dr. Reddy's Laboratories Limited, Bollaram Road Miyapur, Hyderabad 500 049, India, [2]Department of Chemistry, Jawaharlal Nehru Technological University of Anantapur, Anantapur 515002, Andhra Pradesh, India, and [3]Institute of Life Sciences, University of Hyderabad Campus, Gachibowli, Hyderabad 500 046, Andhra Pradesh, India

E-mail: Manojit Pal* - manojitpal@rediffmail.com

*Corresponding author

Experimental Section:

General methods

Unless stated otherwise, reactions were monitored by thin layer chromatography (TLC) on silica gel plates (60 F_{254}), visualized with ultraviolet light or iodine spray. Lipase B from *C. antartica*, Biocatalytics was employed without any previous treatment. Column chromatography was performed on silica gel (60-120 mesh) with distilled petroleum ether and ethyl acetate. ^{1}H and ^{13}C NMR spectra were determined in $CDCl_3$ solution using 400 and 100 (or 125) MHz spectrometers, respectively. Proton chemical shifts (δ) are relative to tetramethylsilane (TMS, δ = 0.0) as an internal standard and expressed in parts per million. Spin multiplicities are given as s (singlet), d (doublet), t (triplet), and m (multiplet) as well as b (broad). Coupling constants (*J*) are given in hertz. Infrared spectra were recorded on a FTIR spectrometer. Melting points were determined by using a Buchi melting point B-540 apparatus. MS spectra were obtained on a mass spectrometer. HRMS was determined using waters LCT premier XETOF ARE-047 apparatus. Chromatographic (HPLC) purity was determined by using the area normalization method and the condition specified in each case: column, mobile phase (range used), flow rate, detection wavelength, and retention times. All the reagents used are commercially available. Before conducting the lipase-catalyzed transesterification reactions, the required analytical methods were established for (a) the HPLC separation of two enantiomers and (b) measurement of enantiomeric excess. In order to avoid the competitive enzymatic hydrolysis of the involved esters, all reagents and solvents were dried prior to use, and a nitrogen atmosphere was employed to ensure low water activity in the reaction medium. The absolute configurations of all compounds were determined by comparison of the sign of the measured specific rotation with those in the literature [1-3].

General procedure for the synthesis of bis-imines (**3**): A mixture of ethane-1,2-diamine (**1**, 5.0 mmol), freshly distilled aldehyde (**2**, 11.0 mmol) and $FeCl_3$ (0.1 mmol) in methanol (40 mL) was stirred at room temperature for the time indicated in Table 1. The reaction mixture was then filtered through a celite bed. The filtrate was collected and concentrated under reduced pressure. The residue obtained was then treated with 10% ethyl acetate - *n*-hexane and filtered to give the required bis-imine.

***(N*^{1}*E,N*^{2}*E)-N*^{1}*,N*^{2}-bis(4-methoxybenzylidene)ethane-1,2-diamine** [4] (**3a**): off-white solid; crystallized from EtOAc-petroleum ether (9:1); mp 109-110 °C; ^{1}H NMR (400 MHz, $CDCl_3$) δ 3.82 (s, 4H), 3.91 (s, 6H), 6.89 (d, *J* = 8.8 Hz, 4H), 7.63 (d, *J* = 8.8 Hz, 4H), 8.20 (s, 2H); ^{13}C NMR ($CDCl_3$, 100 MHz) δ 161.8 (2C, C=N), 161.5 (2C, 2H), 131.9 (4C, C_{aryl}), 129.5 (2C, C_{aryl}), 114.3 (4C, C_{aryl}), 61.6 (2C, CH_2), 55.3 (2C, OCH_3); M/z 297.15; IR (cm^{-1}, KBr) 2843, 1605; HRMS (ESI): calcd for $C_{18}H_{21}N_2O_2$ $(M+H)^+$ 297.1603, found 297.1595.

(*N*^{1}*E*,*N*^{2}*E*)-*N*^{1},*N*^{2}-bis(4-nitrobenzylidene)ethane-1,2-diamine [5] (**3b**): pale yellow solid; mp 196-198 °C; ^{1}H NMR (400 MHz, DMSO-d_6) δ 3.9 (s, 4H), 7.97 (d, *J* = 8.4 Hz, 4H), 8.28 (d, *J* = 8.4 Hz, 4H), 8.50 (s, 2H); ^{13}C NMR (DMSO-d_6, 100 MHz) δ 160.6 (2C, C=N),148.5 (2C, CH), 142.2 (2C, CH), 128.8 (4C, C_{aryl}),123.9 (4C, C_{aryl}), 60.6 (2C, CH_2); M/z 394; IR (cm^{-1}, KBr): 2855, 1603; HRMS (ESI): calcd for $C_{16}H_{15}N_4O_4$ $(M+ H)^+$ 327.1093, found 327.1092.

5,5'-(1*E*,1'*E*)-(ethane-1,2-diylbis(azanylylidene))bis(methanylylidene)bis(2-methoxyphenol) [6] (**3c**): pale yellow solid; crystallized from EtOAc-petroleum ether (90:10); mp 212-214 °C; ^{1}H NMR (400 MHz, DMSO-d_6) δ 3.7 (s, 4H), 3.91 (s, 6H), 6.92 (d, *J* = 4.0 Hz, 2H), 7.03 (d, *J* = 4.0 Hz, 2H), 7.20 (s, 2H), 8.14 (s, 2H), 9.14 (s, 2H); ^{13}C NMR (DMSO-d_6, 100 MHz) δ 161.2 (2C, C=N), 149.9 (2C, C_{aryl}), 146.6 (2C, C-OH), 129.3 (4C, C_{aryl}), 120.8 (4C, C_{aryl}), 60.9 (2C, CH_2), 55.5 (2C, OMe); M/z 329; IR (cm^{-1}, KBr) 3398, 2842, 1639; HRMS (ESI): calcd for $C_{18}H_{21}N_2O_2$ $(M+H)^+$ 329.1501, found 329.1501.

(*N*^{1}*E*,*N*^{2}*E*)-*N*^{1},*N*^{2}-bis(2-fluorobenzylidene)ethane-1,2-diamine [7] (**3d**): off-white solid; crystallized from EtOAc: petroleum ether (90:10); mp 60.8-62.1 °C; ^{1}H NMR (400 MHz, $CDCl_3$) δ 4.00 (s, 4H), 7.15 (m, 2H), 7.36 (m, 2H), 7.53 (m, 2H), 7.95 (m, 2H), 8.6 (s, 2H); ^{13}C NMR ($CDCl_3$, 100 MHz) δ 164.6 (2C, C=N), 159.6 (2C, C_{aryl}), 159.6 (2C,C_{aryl}), 132.2, 132.0 (2C, C_{aryl}), 124.2, 123.6 (2C, C_{aryl}), 115.8, 115.4 (2C, C_{aryl}), 61.8 (2C, CH_2); M/z 272.1; IR (cm^{-1}, KBr) 2857, 1640; HRMS (ESI): calcd for $C_{16}H_{15}N_2F_2$ $(M+ H)^+$ 273.1203, found 273.1204.

4,4'-(1*E*,1'*E*)-(ethane-1,2-diylbis(azanylylidene))bis(methanylylidene)bis(2-methoxyphenol) [8] (**3e**): white solid; crystallized from EtOAc: petroleum ether (90:10); mp 187-189 °C; ^{1}H NMR (400 MHz, DMSO-d_6) δ 3.77 (s, 4H), 3.91 (s, 6H), 6.80 (d, *J* = 7.6 Hz, 2H), 7.09 (d, *J* =

9.6 Hz, 2H), 7.30 (s, 2H), 8.18 (s, 2H), 9.28 (s, 2H); ^{13}C NMR (DMSO-d_6, 100 MHz) δ 161.1 (2C, C=N), 149.9 (2C, C_{aryl}), 146.5 (2C, CH-OH), 129.2 (4C, C_{aryl}), 120.7 (4C, C_{aryl}), 60.9 (2C, CH_2), 55.5 (2C, OMe); M/z 329; IR (cm^{-1}, KBr) 3094, 2866, 1603; HRMS (ESI): calcd for $C_{18}H_{21}N_2O_2$ $(M+H)^+$ 329.1501, found 329.1493.

3,3'-(1*E*,1'*E*)-(ethane-1,2-diylbis(azanylylidene))bis(methanylylidene)diphenol [9] (**3f**): white solid; crystallized from EtOAc: petroleum ether (90:10); mp 167.4-170.5 °C; ^{1}H NMR (400 MHz, DMSO-d_6) δ 3.83 (s, 4H), 6.81 (d, J = 6.0 Hz, 2H), 7.14-7.08 (m, 2H), 7.21 (m, 4H), 8.2 (s, 2H), 9.5 (bs, 2H); ^{13}C NMR (DMSO-d_6, 100 MHz) δ 161.8 (2C, C=N), 157.4 (2C, CH-OH), 137.3 (2C, C_{aryl}), 129.5 (2C, C_{aryl}), 119.2 (2C, C_{aryl}), 117.2 (2C, C_{aryl}), 113.5 (2C, C_{aryl}), 60.9 (2C, CH_2); M/z 269; IR (cm^{-1}, KBr) 3053, 2868, 1632; HRMS (ESI): calcd for $C_{16}H_{17}N_2O_2$ $(M+ H)^+$ 269.1290, found 269.1284.

***(N^{1}E,N^{2}E)-N^1,N^2*-bis(naphthalen-2-ylmethylene)ethane-1,2-diamine** [10] (**3g**): pale yellow solid; crystallized from EtOAc: petroleum ether (90:10), mp 89-91 °C; ^{1}H NMR (400 MHz, $CDCl_3$) δ 4.1 (s, 4H), 6.9 (d, J = 3.6 Hz, 2H), 7.26 (d, J = 3.6 Hz, 4H), 7.82 (m, 6H), 7.92 (d, J = 3.6 Hz, 2H), 8.68 (s, 2H); ^{13}C NMR ($CDCl_3$, 100 MHz) δ 162.7 (2C, C=N), 134.6 (2C, C_{aryl}), 133.8 (2C, C_{aryl}), 133.0 (2C, C_{aryl}), 129.8 (2C, C_{aryl}), 128.5 (2C, C_{aryl}), 128.8 (2C, C_{aryl}), 127.8 (2C, C_{aryl}), 127.1 (2C, C_{aryl}), 126.3 (2C, C_{aryl}), 123.8 (2C, C_{aryl}), 61.7 (2C, CH_2); M/z 337.18; IR (cm^{-1}, KBr) 2868, 1641; HRMS (ESI): calcd for $C_{24}H_{21}N_2$ $(M+ H)^+$ 337.1705, found 337.1707.

***(N^{1}E,N^{2}E)-N^1,N^2*-bis(4-bromobenzylidene)ethane-1,2-diamine** [11] (**3h**): pale yellow solid; crystallized from EtOAc: petroleum ether (90:10); mp 84.3-85.6 °C; ^{1}H NMR (400 MHz, $CDCl_3$) δ 3.96 (s, 4H), 7.25 (d, J = 8.0 Hz, 2H), 7.52 (d, J = 8.0 Hz, 4H), 7.58 (d, J = 7.6 Hz, 2H), 8.2 (s, 2H); ^{13}C NMR ($CDCl_3$, 100 MHz) δ 161.1 (2C, C=N), 138.0 (2C, C_{aryl}),133.5 (2C, C_{aryl}), 130.7 (2C, C_{aryl}), 130.1 (2C, C_{aryl}), 126.9 (2C, C_{aryl}), 122.9 (2C, C_{aryl}), 61.3 (2C, CH_2); M/z 392; IR (cm^{-1}, KBr) 2841, 1642; HRMS (ESI): calcd for $C_{16}H_{15}N_2Br_2$ $(M+ H)^+$ 392.9602, found 392.9600.

***(N^{1}E,N^{2}E)-N^1,N^2*-bis*((1H*-imidazol-2-yl)methylene)ethane-1,2-diamine** (**3i**): light brown solid; crystallized from EtOAc: petroleum ether (90:10); mp 205-213 °C; ^{1}H NMR (400 MHz,

DMSO-d_6) δ 4.1 (s, 4H), 7.81 (d, J = 3.2 Hz, 4H), 8.29 (s, 2H), 12.28 (bs, 2H); ^{13}C NMR (DMSO-d_6, 100 MHz) δ 159.1 (2C, C=N), 137.0 (4C, $C_{imidazolyl}$), 132.0 (2C, $C_{imidazolyl}$), 61.3 (2C, CH_2); M/z 217; IR (cm^{-1}, KBr) 3122, 2836, 1648; HRMS (ESI): calcd for $C_{10}H_{13}N_6$ $(M+ H)^+$ 217.1202, found 217.1200.

***(N^1E,N^2E)-N^1,N^2*-bis(pyridin-2-ylmethylene)ethane-1,2-diamine** (**3j**): off-white solid; crystallized from EtOAc: petroleum ether (90:10); mp 80.1-82.5 °C; ^1H NMR (400 MHz, $CDCl_3$) δ 4.01 (s, 4H), 7.33 (d, J = 9.6 Hz, 2H), 8.07 (d, J = 9.6 Hz, 2H), 8.32 (s, 2H), 8.63 (d. J = 6.4 Hz, 2H), 8.83 (s, 2H); ^{13}C NMR ($CDCl_3$, 100 MHz) δ 159.7 (2C, C=N), 151.5 (2C, $C_{pyridyl}$), 150. 1 (2C, $C_{pyridyl}$), 134.5 (2C, $C_{pyridyl}$), 131.5 (2C, $C_{pyridyl}$), 123.6 (2C, $C_{pyridyl}$), 60.6 (2C, CH_2); M/z 239; IR (cm^{-1}, KBr) 2848, 1644; HRMS (ESI): calcd for $C_{14}H_{15}N_4$ $(M+ H)^+$ 239.1297, found 239.1299.

Synthesis of 3-acetylphenyl ethyl(methyl)carbamate [12,13] **(7):** To a solution of 3-hydroxyacetophenone (3.0 g, 25 mmol) in acetone (30 mL), K_2CO_3 (8.65 g, 62 mmol) and ethyl methyl carbamoyl chloride (6.7 g, 55.37 mmol) were added. The mixture was stirred and heated under reflux for 5 h until complete consumption of the starting material. Then acetone was evaporated under reduced pressure. The resulting residue was dissolved in water and extracted with CH_2Cl_2 (3 × 15 mL). The organic layers were collected, combined, dried over Na_2SO_4, filtered and the solvent was evaporated. The crude product isolated was purified by flash chromatography on silica gel (30% EtOAc/hexane), affording the desired ketone (80% isolated yield); ^1H NMR ($CDCl_3$, 400 MHz) δ 1.26 (m, 3H, CH_3), 2.59 (s, 3H, -$COCH_3$), 3.00 (s, 3H, NCH_3), 3.42 (q, 1H, J = 6.8 Hz), 3.50 (q, 1H, J_1 = 7.2 Hz, J_2 = 6.8 Hz), 7.35 (d, 1H, J = 8.0 Hz), 7.45 (t, 1H, J = 8.0 Hz), 7.69 (s, 1H), 7.77 (d, 1H, J = 7.6 Hz); M/z 221.25 (M^+).

Synthesis of 3-(1-hydroxyethyl)phenyl ethyl(methyl)carbamate [12,13] **[(*RS*)-4]**: To a solution of ketone **7** (2.0 g, 9 mmol) in dry MeOH (25 mL), was added $NaBH_4$ (398 mg, 10.5 mmol) at 0 °C under a nitrogen atmosphere. The mixture was warmed to room temperature and stirred for 1 h. The excess and unreacted hydride was destroyed by the careful addition of water (5 mL). MeOH was then evaporated and the mixture extracted with CH_2Cl_2 (3 x 15 mL). The organic layers were collected, combined, dried over anhydrous Na_2SO_4,

filtered and the solvent was evaporated under reduced pressure. The crude reaction mixture was purified by flash chromatography on silica gel (10% EtOAc/hexane) to afford the racemic alcohol **3** as colourless oil (95% isolated yield); ^{1}H NMR ($CDCl_3$, 400 MHz) δ 1.26 (m, 3H, CH_3), 1.50 (d, 3H, J = 6.4 Hz, CH_3), 3.07 (s, 3H, CH_3), 3.43 (q, 1H, J_1 = 7.2 Hz, J_2 = 6.8 Hz), 3.48 (q, 1H, J_1= 6.8 Hz, J_2 =7.2 Hz), 4.9 (q, 1H, J_1 = 6.8 Hz, J_2 = 6.0 Hz), 7.01 (d, 1H, J = 8.0 Hz), 7.18 (d, 1H, J = 13.6 Hz), 7.26 (s, 1H), 7.33 (t, 1H, J = 8.0 Hz); M/z 224.20 (M+1); HPLC: 99%, [(*S*)-isomer: 48%, (*R*)-isomer: 51%] column: Chiralpak IC (250 x 4.6 mm, 5.0 µm), mobile phase A: 0.05% TFA in water, mobile phase B: n-hexane: IPA (80:20), concentration: 0.5 mg/mL, diluents: ethanol, run time: 30.0 min., temperature: 25 °C; flow: 1.0 mL/min, UV: 220 nm, retention time: (*S*)-alcohol: 8.7 min; (*R*)-alcohol: 9.3 min.

Typical procedure for the enzymatic kinetic resolution of (*RS*)**-4**: To a suspension of a bis-imine **3i** (0.065 g, 0.3 mmol) in vinyl acetate (20 mL), anhydrous cobalt chloride (0.040 g, 0.3 mmol) was added and the mixture stirred for 1 h. To this was added racemic alcohol (*RS*)**-4** (1.0 g, 4.4 mmol) and CAL-B (150 mg) under a nitrogen atmosphere. The mixture was shaken @ 250-300 rpm at 30 °C. An aliquot was collected after 8 h and analyzed by HPLC which indicated an ee of 91% with respect to the substrate. The reaction was continued for an additional 4 h and then terminated by separating the enzyme via filtration. The filtrate was evaporated and the crude product was purified by flash chromatography on silica gel (10-15% EtOAc/hexane), affording the acetate (*R*)**-5** [80% isolated yield and >99% ee] and the alcohol (*S*)**-4** [84% isolated yield and >99% ee].

Data [1-3] of alcohol (*S*)**-4**: ^{1}H NMR ($CDCl_3$, 400 MHz) δ 1.26 (m, 3H, CH_3), 1.50 (d, 3H, J = 6.4 Hz, CH_3), 3.07 (m, 3H, CH_3), 3.43 (q, 1H, J_1 = 7.2 Hz, J_2 = 6.8 Hz), 3.48 (q, 1H, J_1 = 6.8 Hz, J_2 = 7.2 Hz), 4.9 (t, 1H, J_1 = 6.8 Hz, J_2 = 6.0 Hz), 7.01 (d, 1H, J = 8.0 Hz), 7.18 (d,1H, J = 8.0 Hz), 7.26 (s, 1H), 7.33 (t, 1H, J = 8.0 Hz); ^{13}C NMR ($CDCl_3$, 100 MHz) δ 154.4 (1C, C=O), 151.6 (C_{aryl}), 129.3 (C_{aryl}), 124.7 (C_{aryl}), 121.2 (C_{aryl}), 120.8 ($2C_{aryl}$), 69.0 (1C), 43.4 (1C, CH_2 of carbamoyl), 34.6 (1C, $Me_{carbamoyl}$), 20.3 (1C, CH_3), 12.4 (1C, $Me_{carbamoyl}$),; IR (cm^{-1}, KBr) 3440, 1709 (C=O); HPLC: > 99.0 %, column: chiralpak IC (250 x 4.6 mm, 5.0 µm), mobile phase A: 0.05% TFA in water, mobile phase B: *n*-hexane: IPA (80:20), concentration: 0.5 mg/mL, diluents: ethanol, run time: 30.0 min., temperature: 25 °C; flow: 1.0 mL/min, UV: 220

nm, retention time: (*S*)-alcohol: 8.7 min; HRMS (ESI): calcd for $C_{12}H_{17}NO_3$ $(M+H)^+$ 224.1287, found 224.1293; $[\alpha]^{20}_D$ = -22.96 (C=1, $CHCl_3$).

Data of acetate (*R*)-**5**: ^{1}H NMR ($CDCl_3$, 400 MHz) δ 1.26 (m, 3H, CH_3), 1.50 (d, 3H, J = 6.4 Hz, CH_3), 1.99 (s, 3H, CH_3), 3.07 (m, 3H, CH_3), 3.43 (t, 1H, J_1 =7.2 Hz, J_2 = 6.8 Hz), 3.48 (t, 1H, J_1 = 6.8 Hz, J_2 = 7.2 Hz), 4.9 (t, 1H, J_1 = 6.8 Hz, J_2 = 6.0 Hz), 7.01 (d, 1H, J = 8.0 Hz), 7.18 (d, 1H, J = 13.6 Hz), 7.26 (s, 1H), 7.33 (t, 1H, J = 8.0 Hz); ^{13}C NMR ($CDCl_3$, 100 MHz) δ 170.1 (1C, C=O), 154.4 (1C, C=O), 151.6 (C_{aryl}), 129.3 (C_{aryl}), 124.7 (C_{aryl}), 121.2 (C_{aryl}), 120.8 (2C_{aryl}), 69.0 (1C), 44.0 (1C, CH_2-carbamoyl), 34.6 (1C, $Me_{carbamoyl}$), 22.1 (1C, CH_3), 21.3 (1C, CH_3), 13.6 (1C, rotamer), 12.4 (1C, $Me_{carbamoyl}$); IR (cm $^{-1}$, KBr) 2979, 1721 (C=O); $(M+NH_4)^+$: 288.32; HPLC: > 99.0 %, Column: Chiralpak IC (250 x 4.6 mm, 5.0 µm), mobile phase A: 0.05% TFA in water, mobile phase B: n-hexane: IPA (80:20), concentration: 0.5 mg/mL, diluents: ethanol run time: 30.0 min.; temperature: 25 °C; flow: 1.0 mL/min, UV: 220 nm., retention time: 10.4 min; HRMS (ESI): calcd for $C_{14}H_{19}NO_4$ $(M+H)^+$ 266.1392, found 266.1380; $[\alpha]^{20}_D$ = 61.73 (C=1, $CHCl_3$).

Hydrolysis of acetate (*R*)-**5**: To a suspension of (*R*)-**5** (0.1 g) in methanol (2 mL), 3N HCl (3 mL) was added at room temperature. The temperature of the mixture was raised to 40-45 °C, maintained at this temperature for 30 min, and the absence of starting material monitored by TLC. The reaction was then stopped and the mixture basified with 1N NaOH until neutral, and extracted with CH_2Cl_2 (3 × 5 mL). The organic layers were collected, combined and concentrated. The residue was purified by flash chromatography on silica gel (10% EtOAc/hexane) affording the alcohol [12] (*R*)-**4** [70% isolated yield and > 99% ee], $[\alpha]^{20}D$ = 23.5 (c = 1.0, CH_3Cl). HPLC: > 99.0 %, column: chiralpak IC (250 x 4.6 mm, 5.0 µm), mobile phase A: 0.05% TFA in water, mobile phase B: *n*-hexane: IPA (80:20), concentration: 0.5 mg/mL, diluents: ethanol, run time: 30.0 min., temperature: 25 °C, flow: 1 mL / min, UV: 220 nm., retention time: 9.9 min.

Synthesis of (*S*)-3-[1-(dimethylamino)ethyl]phenyl ethyl(methyl)carbamate [12,13] [(*S*)-**8**]**:** To a suspension of (*R*)-**5** (400 mg, 1.0 mmol) in toluene, dimethylamine (24 wt % in toluene, 4.0 mL, 15 mmol) was added at 5-10 °C, maintained at the same temperature for

20-24 h, and the absence of starting material was monitored by TLC. The reaction mixture was partitioned between toluene and water. The organic layer was collected, washed with 10% sodium chloride solution, and concentrated under vacuum. The residue was purified by column chromatography (EtOAc/hexane/TEA 10/90/0.1) affording 225 mg of (*S*)-rivastigmine as a light yellow oil (60% isolated yield). ^{1}H NMR ($CDCl_3$, 300 MHz) δ 1.17-1.27 (m, 3H), 1.37 (d, 3H, J = 6.4 Hz, CH_3), 2.21 (s, 6H), 3.04 (s, 3H, CH_3), 3.25 (q, J_1 = 7.2 Hz, J_2 = 6.4 Hz), 3.43 (q, 1H, J_1 = 7.2 Hz, J_2 = 6.8 Hz), 3.48 (q, 1H, J_1 = 6.8 Hz, J_2 = 7.2 Hz), 7.01 (d, 1H, J = 8.0 Hz), 7.18 (d, 1H, J = 8.0 Hz), 7.26 (s, 1H,), 7.33 (t, 1H, J = 8.0 Hz); ^{13}C NMR ($CDCl_3$, 100 MHz) δ 154.4 (1C, C=O), 151.4 (CH), 129.3 (CH), 124.7 (CH), 121.2 (CH), 120.8 (2C, CH), 77.1 (1C), 66.0 (1C, CH_2), 43.9 (2C, N-Me), 34.6 (1C, $Me_{carbamoyl}$), 20.3 (1C, CH_3), 12.4 (1C, $Me_{carbamoyl}$); M/z 251.20 (M+ H)$^+$; IR (cm^{-1}, KBr) 2975, 1723 (C=O); HRMS (ESI): calcd for $C_{14}H_{22}N_2O_2$ (M+ H)$^+$ 251.1760, found 251.1767; $[\alpha]^{20}_D$ = -33.90 (C=1, $CHCl_3$).

References

1. Naemura, K.; Murata, M.; Tanaka, R.; Yano, M.; Hirose, K.; Tobe, Y. *Tetrahedron: Asymmetry* **1996,** *7,* 3285;
2. Yadav, J. S.; Nanda, S.; Reddy, P. T.; Rao, A. B. *J. Org. Chem.* **2002,** *67,* 3900;
3. Kim, M.-J.; Kim, H. M.; Kim, D.; Ahn, Y.; Park, J. *Green Chem.* **2004,** *6,* 471.
4. Kadam, A. J.; Mane, R. B. *Indian J. Chem. Sec. B* **2000,** *39B,* 628.
5. Reyes-Arellano, A.; Vega-Ramirez, L.; Najera-Mundo, J. A.; Salgado-Zamora, H.; Molins, E.; Peralta-Cruz, J.; Tamariz, J. *J. Molecular Structure* **2003,** *655,* 141.
6. Galey, J. B.; Dumats, J. PCT Int Appl. No (1994), WO 9411338.
7. Bergman, J.; Brynolf, A. *Tetrahedron Lett.* **1989,** *30,* 2979.
8. Ghose, B. N. *J. Chem. Engineering Data* **1984,** *29,* 237.
9. Mahon, M. F.; McGinley, J.; Rooney, A. D.; Walsh, J. *Inorganica Chimica Acta* **2009,** *362,* 2353.
10. Liu, H.; Zhang, H.-L.; Wang, S.-J.; Mi, A.-Q.; Jiang, Y.-Z.; Gong, L.-Z. *Tetrahedron Asymm* **2005,** *16,* 2901.
11. Fun, H.-K.; Mirkhani, V.; Kia, R.; Vartooni, A. R. *Acta Crystallographica, Sec E* **2008,** *E64,* 1374.

12. Gaitonde, A.; Mangle, M.; Pawar, S. R. Process for the preparation of aminoalkyl phenylcarbamates. World patent application no. WO2005/061446, July 7, 2005.
13. Fieldhouse, R. Process for the preparation of tertiary amines attached to a secondary carbon centre. World patent application no. WO2005/058804, June 30, 2005.
